# Hunger and Satiety Gauge Reward Sensitivity

**DOI:** 10.3389/fendo.2017.00104

**Published:** 2017-05-18

**Authors:** Ryan Michael Cassidy, Qingchun Tong

**Affiliations:** ^1^Brown Foundation of the Institute of Molecular Medicine for the Prevention of Human Diseases of McGovern Medical School, Neuroscience Program MD Anderson Cancer Center and UTHealth Graduate School of Biological Sciences, The University of Texas Health Science Center at Houston, Houston, TX, USA

**Keywords:** hunger, satiety, obesity, hypothalamus, reward, superstimulus, dopamine

## Abstract

Many of the neurocircuits and hormones known to underlie the sensations of hunger and satiety also substantially alter the activity of the dopaminergic reward system. Much interest lies in the ways that hunger, satiety, and reward tie together, as the epidemic of obesity seems tied to the recent development and mass availability of highly palatable foods. In this review, we will first discuss the basic neurocircuitry of the midbrain and basal forebrain reward system. We will elaborate how several important mediators of hunger—the agouti-related protein neurons of the arcuate nucleus, the lateral hypothalamic nucleus, and ghrelin—enhance the sensitivity of the dopaminergic reward system. Then, we will elaborate how mediators of satiety—the nucleus tractus solitarius, pro-opiomelanocortin neurons of the arcuate nucleus, and its peripheral hormonal influences such as leptin—reduce the reward system sensitivity. We hope to provide a template by which future research may identify the ways in which highly rewarding foods bypass this balanced system to produce excessive food consumption.

## Introduction

In evolutionary psychology, a supernormal stimulus or “superstimulus” is some evolutionarily novel concentration of engaging characteristics, which produces a stronger response than the natural one (1). As Pinker described it, strawberry cheesecake is a superstimulus as compared to a Neolithic human diet; it overloads the senses and drives caloric overconsumption, combining the “sweet taste of ripe fruit, the creamy mouth feel of fats and oils from nuts and meat, and the coolness of fresh water” (2). The debate is ongoing as to what exactly differentiates a superstimulus from a regular one or whether it is truly maladaptive to create them or seek them out (1). Nevertheless, an important concept for neuroscience emerges from this discussion; certain systems governing the reaction to rewarding stimulus can be overloaded and their negative feedback component overridden. This hypothesis provides explanation for the panoply of excessive behaviors we cope with as a society, often conceptualized as “behavioral addictions”: drug addiction, internet addiction, porn addiction, food addiction, and so on (3). Even physical activity in some individuals meets the criteria for behavioral addiction, demonstrating the complex nature of this phenomenon (4). It is necessary to provide biological evidence for the existence of the balanced system which these superstimuli overload.

The neurocircuitry and endocrinology underlying hunger and satiety may represent the best studied system in this regard. It clearly produces a physiological balance in certain conditions with regular stimulus, such as a laboratory mouse fed rodent chow its whole life producing a normal weight, and is dysregulated in other conditions that contain superstimuli, such as when that mouse is fed a high-fat, high-carbohydrate diet producing obesity. The signals encoding hunger and satiety alter the brain’s dopaminergic reward system in a multitude of ways. In this review, we will discuss the mechanisms by which these signals, primarily hypothalamic neurocircuits and neuropeptides in combination with peripheral hormones, modulate midbrain dopaminergic activity to alter reward salience and value. By laying out this evidence, we provide a substrate for future research to examine how superstimulus foods, such as cheesecake or high-fat/high-carbohydrate chow, drive so-called hedonic feeding and produce obesity.

The relationship of the midbrain dopaminergic reward system and hypothalamic neurocircuits governing hunger and satiety, hereafter referred to as the *reward system* and *hunger system*, is ancient. The patterned expression of genes necessary to produce segmentation of the brain at the mesencephalon (midbrain) and diencephalon (hypothalamus) occurred very early in chordate evolution (5, 6). Both dopamine neuronal receptors and hypothalamic feeding-related peptides and their associated receptors are present in most vertebrates and have similar functions across taxa (7–9). Given this intimate association, it is not surprising that they share a fundamental interdependence. For example, dopamine-deficient mice stop feeding a few weeks after birth; administration of l-DOPA reverses this phenotype and restores normal growth (10). Conversely, knockout of orexin, a hypothalamic neuropeptide associated with hunger, reduces dopamine response to cocaine (11). They also produce cross-sensitization or desensitization; food-restricted, hungry mice have enhanced response and reinforcement to amphetamine or cocaine (drugs which flood the brain with dopamine), and satiety signals such as leptin reduce the drive to seek self-administration of these drugs (12, 13). As will be discussed below, hypothalamic and endocrine components of the hunger system alter the activity of the reward system. To demonstrate this, we will first present a brief overview of the neurocircuitry of the reward system. Then, we will chart the various ways the hunger system interacts with the reward system.

## Neurocircuitry of the Reward System

### The Ventral Tegmental Area (VTA)

The VTA and substantia nigra pars compacta (SNc) are immediately caudal to the posterior hypothalamus, brace the third ventricle, and contain the major source of dopaminergic outflow to the rest of the brain. The SNc is best known for its role in the nigrostriatal pathway regulating the dorsal striatum in movement, and the VTA for mediating salience, motivation, and reward and aversion-related learning (14, 15). Salience refers to the attention paid to the stimulus; an increase in salience means the stimulus, if identified, will be more likely to draw the organism’s attention. The value of the stimulus, whether it is rewarding or aversive, refers to whether a stimulus induces behavior to acquire it or avoid it, respectively (15). Rewarding stimuli produce a positive valence when acquired and a negative valence when unable to be acquired; the converse is true with aversive stimuli (15).

The VTA dopaminergic neurons are the primary mediators of the behavioral response to a rewarding or aversive stimulus (16). They are not uniform in their activity or projection targets, and thus activation of one neuron may produce substantially different behavioral output than another. This is why studies evaluating the rewarding nature of dopamine often focus on the VTA to nucleus accumbens (NAc) projections specifically; this will be discussed below. However, much effort has been spent elucidating the ways in which local VTA dopaminergic neurons encode reward across brain regions by alteration in firing pattern, increase or decrease in action potential frequency, and projection target. The literature is incomplete on this topic, but discussion of some of these mechanisms sets the stage for further discussion of how the hunger system interfaces with the VTA dopaminergic neurons. For example, *in vivo* recording of the VTA during a conditioned place preference task suggests that one subset of dopaminergic neurons exhibits phasic activation in response to reward-related cues or reward consumption; another exhibits phasic inhibition in response to aversive stimulus or the absence of reward consumption after a reward cue (17). Tonic activation of dopaminergic neurons can produce the opposite effect of phasic activity on the same target and will decrease reward consumption (18). Thus, a given projection target receives either increased or decreased dopamine input dependent on the valence of the reward. Furthermore, dopaminergic neurons vary in their projection targets; the VTA’s projections are heterogeneous. Dependent on the projection target, an increase in dopamine outflow produces either rewarding or aversive responses (14, 19). As has been well-established, VTA projections to the NAc core (NAcc) and NAc shell (NAcSh) increase dopamine release in response to a rewarding stimulus and induce goal-direct behavior to acquire and consume it (14). Conversely, VTA dopaminergic neurons projecting to the medial prefrontal cortex are activated in response to an aversive stimulus and produce aversive behaviors (19). However, even within the same target, dopaminergic activation can code both types of behaviors; VTA dopaminergic projections to the lateral portion of the NAcSh are activated in response to both rewarding and aversive stimulus (19).

The VTA also possesses neurons releasing the classic neurotransmitters glutamate and GABA. The function of these glutamatergic and GABAergic neurons is less well-known, but recent evidence indicates they also participate in valence-related responses. VTA glutamatergic projections to the lateral habenula (LHb) play a significant role in encoding aversive learning (20). VTA glutamatergic projections to the NAcSh act in concert with the dopaminergic projections to produce reward-mediated behavior (14). Finally, VTA GABAergic neurons projecting to the LHb appear to inhibit this area to enhance positive valence responses (21). Recent analysis has identified that some VTA neurons corelease glutamate and dopamine—it is as yet unknown whether this occurs at the same synapse or at separate synaptic targets (14). Further research is needed to fully evaluate how the classic fast-acting neurotransmitters coordinate with dopaminergic neurons to produce the full suite of valence-related behaviors and alter future learned responses.

### The NAc

The NAc is part of the ventral striatum and extended amygdala in the basal forebrain, and it mediates much of the motivated behavior produced in response to VTA dopaminergic outflow after sensation of a rewarding stimulus. Many components of the hunger system act here as well as in the VTA to alter the responsiveness to rewarding stimulus; thus, some description of its components and basic activity follows. The NAc is divided into a medial shell (NAcSh) and lateral core (NAcc). Self-administration of cocaine into the NAcSh is highly rewarding and rapidly produces cue-responsiveness with locomotor sensitization to anticipation of the drug (22–24). Self-administration of cocaine into the NAcc, however, is not reinforcing (22). Phasic activity of VTA dopaminergic projections to the NAcc instead responds to risk and prediction error in response to reward presentation (22, 24, 25). Thus, a basic paradigm can be constructed, where the NAcc responds to the salience, availability, and risk of acquiring the reward to produce motivation to pursue it, and the NAcSh responds to the positive valence of the reward acquisition, learns the cues which associate with the reward, and enhances the future salience of those cues. Interestingly, if dopamine is depleted in the NAc but reward acquisition is low effort, rats will still take the reward; however, if it requires high effort, rats will choose less effort-requiring behaviors (26). Thus, the level of dopamine in the NAc may provide a rough proxy for the amount of motivation an animal has to ignore risk and effort costs of acquiring a reward.

Both the shell and the core are inhibitory on all downstream targets; the vast majority of neurons are the GABAergic medium spiny neurons (MSNs). These are divided by receptor profile. There are D1R-MSNs, possessing excitatory D1R-like dopamine receptors (D1R and D5R), and D2R-MSNs, possessing inhibitory D2R-like dopamine receptors (D2R, D3R, and D4R). A significant minority express both receptor subtypes (27). The projection fields of the NAcSh and NAcc are wide and differ from each other in several important respects for their mediation of behavior. The NAcSh densely projects to the ventromedial ventral pallidum, lateral hypothalamic area (LH), and lateral preoptic area, whereas the NAcc projects to the dorsolateral ventral pallidum, subthalamic nucleus, and substantia nigra pars reticulata (22). The NAcSh shares significant reciprocal connections with feeding-related areas of the hypothalamus, whereas the NAcc primarily interacts with the basal ganglia. Thus, the NAcSh responds more to signals from the hunger system than the NAcc and will feature more prominently in this discussion.

## Hunger Neurocircuits Select for Increased Reward System Activity in the Presence of Food

**Figure 1:**
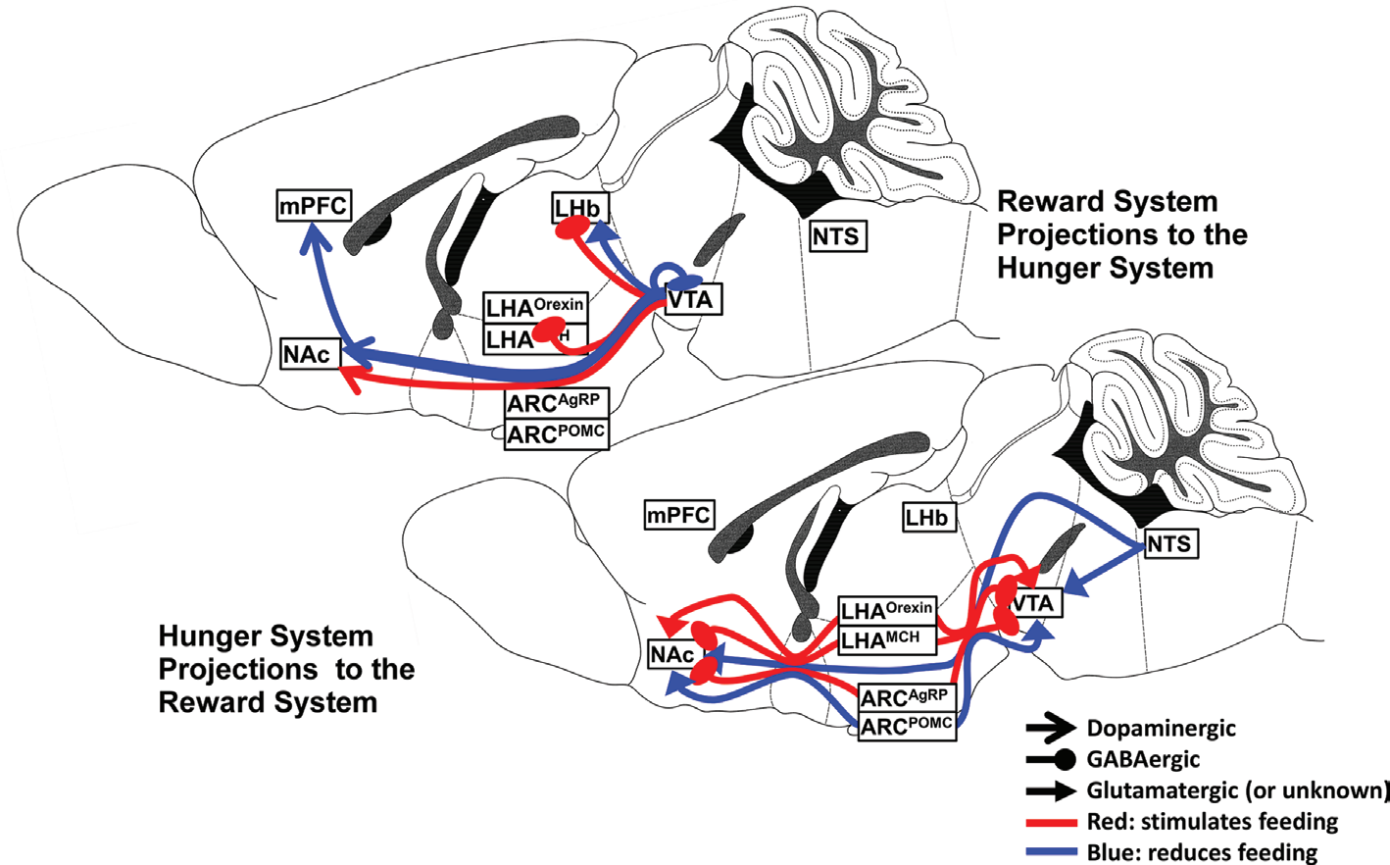
**Hunger and satiety neurocircuits interface with the midbrain–basal forebrain reward pathway**.

There are numerous and dense interconnections between the hypothalamic nuclei, VTA, and NAc, and a wealth of neuropeptide and neurocircuit data exists to support the powerful influence of the several hypothalamic nuclei and their specific neuronal subtypes on the reward system. The interaction is complex and dynamic, depending upon both the availability of food and the endocrine manipulation of the system, as will be discussed later. A summary of the major hunger and satiety neurocircuits influencing the reward system is shown in Figure 1.

### Arcuate Nucleus

The arcuate nucleus of the hypothalamus (Arc) sits adjacent to the third ventricle immediately ventral to the paraventricular hypothalamic nucleus (PVH). These two nuclei share the distinction of integrating central nervous system (CNS) input into the hypothalamic–pituitary axis and are the major source of the “releasing hormones,” which are secreted into the hypophyseal portal veins to alter anterior pituitary production of various hormones. Thus, the Arc possesses a multitude of neuronal populations defined by their neuropeptide content, such as gonadotropin-releasing hormone neurons, growth hormone releasing hormone neurons, kisspeptin neurons, tuberoinfundibular dopamine (TIDA) neurons regulating prolactin release, somatostatin neurons, and so on. Many of these neurons alter feeding behavior, but their interaction with the dopaminergic reward system is poorly understood at this point in time (28). Thus, our discussion will focus on the agouti-related protein (AgRP)/neuropeptide Y (NPY) neurons that govern hunger and TIDA neurons role in feeding behavior. The pro-opiomelanocortin (POMC) neurons that govern satiety will be discussed later.

#### AgRP/NPY-Expressing Neurons

The AgRP/NPY-expressing neurons are found solely within the Arc (29). NPY acts on NPY receptors (Y1, Y2, Y4, and Y5 which are G_i_PCRs) (30). AgRP is an inverse agonist of melanocortin receptors (MCRs; MC3R and MC4R, which are G_s_PCRs and MAPK pathway activators) (31–35). Perhaps because of this, these neurons share with POMC neurons the same set of connections with hypothalamic and extrahypothalamic nuclei (29, 36). Thus, AgRP neurons are strong inhibitors of their downstream targets *via* GABA release, inverse agonism of MC-Rs, and NPYR-Gi activity. These neurons respond to a wide array of peripheral and central signals of energy balance, such as leptin, ghrelin, low glucose concentration, and gustatory sensation, and are activated during fasting (37–41). Surprisingly, given this role, knockout of AgRP by itself or in combination with NPY does not produce any obvious phenotype either in *ad libitum* or starvation feeding conditions—only in old age do they demonstrate slightly reduced body weight and adiposity due to increased metabolic rate (42, 43). Furthermore, neonatal destruction of the AgRP/NPY neurons has minimal effect on feeding; only adult ablation of these neurons prevents feeding behavior and leads mice in this condition to starve to death (44–46). Interestingly, several AgRP/NPY neuronal projections are not formed until a week postnatal in mice, such as to the PVH; there are many opportunities for developmental compensation to alter the neonatal AgRP/NPY ablation phenotype, which deserve further study to understand the homeostasis of feeding behavior (47). Much recent effort has been spent on understanding the acute dynamics of AgRP/NPY neuronal activity in hunger and reward.

Optogenetic stimulation produces food seeking and food consuming behaviors, with enhanced risk-taking and reduced anxiety (48–51). Their activity is aversive, as mice avoid the side of a chamber associated with their optogenetic activation (52). AgRP neurons select for food consumption; when activated, they reduce motivation to engage in other behaviors such as social interactions or drinking water when thirsty (51). Sustained AgRP neuronal activity is not necessary to produce feeding, and *in vivo* recording demonstrates that these neurons stop firing in the presence of food cues (53). Further optogenetic evidence demonstrates that a brief period of activation, prior to presentation with any food stimulus, will produce subsequent feeding, enhanced motivation to work for food, and selection for calorie dense foods (54). As strength of AgRP signal increases, there is a first-order kinetic increase in length of feeding and motivation to work which saturates (54). Stimulating AgRP projections to the PVH, bed nucleus of the stria terminalis, or LH are all individually sufficient to produce this effect (54). However, AgRP neurons also synapse on the VTA and regulate the reward system through this connection. AgRP projections to the VTA inhibit dopaminergic and glutamatergic release in the NAc and reduce the development of long-term potentiation (LTP) (55). It can be argued that AgRP neurons in the Arc reduce activity of the reward system while activating the hunger system, priming it to respond to food and not other stimuli; once food is spotted, cessation of AgRP neuronal activity releases the brakes on the reward system to enhance dopaminergic outflow. The increase in dopamine in the NAc likely increases the willingness to work for food and take risks to acquire it.

#### TIDA Neurons

The dopaminergic neurons of the Arc regulate the release of prolactin from the anterior pituitary. A subset of TIDA neurons appear to be functionally distinct from governing prolactin; these corelease GABA and dopamine, and deletion of prolactin receptor within this subpopulation has no effect on prolactin secretion regulation (56). A recent study demonstrated that optogenetic stimulation of these neurons produces feeding behavior independent of their stimulation of prolactin release, and inhibition of these neurons reduces body weight (57). Activation of these neurons, which are ghrelin sensitive, inhibited POMC neurons and excited AgRP/NPY neurons. While these are not considered part of the dopaminergic reward system, their role in detecting the intersection of hunger and reward is relevant to this discussion as any manipulation altering whole-brain dopamine systems (such as dopamine reuptake inhibitors such as cocaine) will alter the activity of these neurons and may subtly alter the phenotype.

### The Lateral Hypothalamus

One of the major downstream targets of AgRP neurons is the LH. This was classically understood as both a hunger center and reward hot spot from early lesion and electrical stimulation studies. The LH has extensive projections divided by multiple subpopulations of neurons that express various neuropeptides as well as solely fast-acting neurotransmitter neurons (58). Many of these fast-acting projections play a role in mediating hunger and reward. LH glutamatergic projections to the LHb prevent consumption of a conditioned reward of sucrose and has negative valence (59). Inhibition of this same projection has positive valence and induces sucrose consumption. Conversely, LH GABAergic projections appear to mediate consumption; activation of LH GABAergic neurons produces consumption regardless of the target’s food value, such as ethanol, water, saccharin, sucrose, or wood (60). The projection targets of the LH GABAergic neurons may relate to different aspects of this behavior. For example, LH GABAergic projections to the PVH produce directed food consumption (61). However, activation of the LH-VTA GABAergic projections produce non-directed gnawing and licking of immediately available objects, despite the availability of a sucrose reward distant from the mouse (62). These LH-VTA GABAergic neurons inhibit both a subpopulation of dopaminergic and GABAergic neurons within the VTA. Interestingly, the reciprocal VTA-LH projection is observed to be activated in response to reward omission; the disorganized feeding behavior from LH-VTA projection may occur because of the lack of this feedback information (62). Indeed, a recent study evaluating lateral septum inhibitory influence on the LH found that one subset of LH GABAergic neurons are activated during food approach and another during food consumption, indicating that a temporal sequence of GABAergic subpopulation activation occurs to produce successful food consumption (63).

#### Orexin Neurons

Another reciprocal functional relationship between LH neuronal subtypes can be found in orexin (hypocretin) and melanin-concentrating hormone (MCH) neuropeptide-expressing neurons. Orexin, as the name suggests, is an orexigenic or food consumption-inducing neuropeptide released primarily by glutamatergic neurons in the medial and dorsal portions of the LH. Orexin A and B are co-released from the same neurons and bind to their receptors O1R and O2R, potently inducing arousal *via* G_s_ signaling. Orexin release dramatically increases in release in the human amygdala upon waking up or during arousing positive stimuli such as laughing or talking (64). Orexinergic neurons are activated in response to learned reward cues, but not novel objects; for example, activating them can reinstate extinguished drug-seeking behavior (65). Indeed, O2R protein levels in the NAc are elevated for up to 60 days after discontinuation of repeated cocaine administration (66). They are depolarized in response to low glucose, and directly activate VTA dopaminergic neurons which project to both the medial and lateral portions of the NAcSh (67). Orexin receptors also exist within both of these subdivisions; injection of Orexin A into the medial NAcSh induces a sucrose pleasure-associated facial response, whereas injection into the lateral NAcSh induces a sucrose seeking reaction (68). O1R activation increases NMDA glutamate receptor activity, a sign of LTP formation, whereas blockade of O1R in the VTA reduces the rate of self-administration of cocaine or chocolate (11, 69). Thus, orexinergic neurons clearly enhance the seeking and acquisition of a learned reward, partially *via* activation of the dopaminergic pathway as well as potentially enhancing the reinforcing quality of those rewards.

#### MCH Neurons

Melanin-concentrating hormone neurons are released from the anatomically adjacent dorsolateral LH and have a complex reciprocal relationship with orexin neurons. They linked with induction of sleep and directly inhibit orexin neurons *via* the MCH receptor (MCH1R) G_i_ protein coupled signaling (70). Their interaction with feeding behavior is complex. Knockdown of MCH1R produces hyperphagia, hyperactivity with increased foraging behaviors; however, these mice have a baseline lower body weight than controls, gain less weight on a high-fat diet, and exhibit an increased metabolic rate (71, 72). Elevation of glucose concentration from fasting levels to fed-state levels inhibits orexin neurons and depolarizes MCH neurons. It appears that MCHergic activity appears to play a role in placing a brake on orexin-induced seeking and consuming behaviors after food is acquired (73). However, injection of MCH into the lateral ventricle increases food consumption, without producing long-term weight gain (74). It appears that MCH neurons integrate olfactory, taste, and gut sensory input about the nutritional value of food and project to the VTA, NAc, and dorsal striatum in order to enhance the rewarding value of nutritionally valuable foods (75, 76). To this end, optogenetic stimulation of MCH neurons increases dopamine levels in the striatum only when paired with active consumption of an artificial sweetener; without stimulation, only caloric foods like sucrose induce this dopamine release (76). Pairing these stimuli produced a future preference for the artificial sweetener over sucrose—opposite of the control mouse preference. As further evidence of this, ablation of MCH neurons prevented the natural spike of dopamine in the striatum after the consumption of sucrose—though, interestingly, it did not ablate preference for sweet flavor over water, indicating that other mechanisms are at play (76). Thus, MCH neuronal activity increases when olfaction, taste, and gut nutrient sensors indicate that the food under active consumption is calorically valuable; it enhances the rewarding value of food by increasing VTA dopaminergic activity in the NAc.

The pattern of MCH1R activation within the NAc is similarly complex and deserves some further discussion. These receptors are coexpressed with D1R and D2R on opioid-producing MSNs. Activation of MCH1R here induces feeding and causes a depressed phenotype on the forced swim test, implying reduced locomotor drive (77, 78). Given that MCH1R is inhibitory, it is unsurprising that MSNs have decreased membrane excitability and reduced AMPA glutamatergic receptor currents; furthermore, antagonism of MCH1R reduces cocaine self-administration or cue-induced reinstatement of cocaine seeking behavior. However, on MSNs which coexpress D1R, D2R, and MCH1R, there is a unique synergy which enhances their firing activity and activates a phosphorylation cascade known to increase NMDA receptor activation (79). Some of this response is opioid dependent, as blockade of any of the three opioid receptors in the NAcSh prevents MCH-induced facial pleasure expression in response to oral gavage of sucrose (80). It appears that MCH1R activity within the NAc may shift the reward system response to enhance immediate food consumption and learning the nutrient value of food and dampen the seeking function of the reward system.

### Hunger Enhances Sensitivity to Reward

The above discussion details how several neuronal populations—AgRP/NPY neurons, LH GABAergic neurons, orexin neurons, and MCH neurons—each alter the activity of the reward system in a distinct way as part of their contribution to the sensation of hunger. AgRP neurons reduce reward system sensitivity and inhibit its function until food is detected. The food cue silences their activity, disinhibiting the VTA and NAc to produce a large increase in dopamine release. This increases both the salience and value of the food cue. A subset of LH GABAergic neurons act in concert with orexin neurons to respond to these cues and produce food-seeking behavior, enhancing VTA dopaminergic release into the NAc. Other LH GABAergic neurons act with MCH neurons to also enhance the rewarding value of food and increase learning of nutritionally valuable food-related cues, producing food consumption. Once this process begins, satiety neurocircuits begin to act in order to limit overconsumption, as will be detailed below.

## Satiety Neurocircuits Decrease the Activity of the Reward System

The neurocircuitry of satiety is not as well-known as that of hunger. The general paradigm appears to be as follows: peripheral signals of positive energy balance, primarily hormonal, travel to the brain to inhibit the activity of hunger-producing neurocircuits. However, three populations of neurons within the CNS, defined by their neuropeptide content, are activated by these signals and directly influence the reward system. These are the POMC neurons of the Arc, the POMC neurons of the nucleus tractus solitarius (NTS), and the preproglucagon (PPG) neurons of the NTS. The NTS neurons integrate peripheral satiety signals, such as leptin, cholecystokinin (CCK), glucagon-like peptide 1 (GLP1), and gut distention, to induce rapid satiety. The role of POMC neurons is more complex, but appears to reduce the immediate value of the food reward while maintaining future responsiveness to that same reward.

### PPG Neurons in NTS

The NTS has multiple functions both related to primary routing of taste information as well as being the routing point for significant amounts of autonomic and peripheral hormonal information entering the brain. It is situated in the dorsal motor vagal nerve complex and receives the vagal sensory afferents. These afferents are activated by GLP1, CCK, and leptin. The first two are released from stomach and intestinal cells and leptin from adipocytes in response to increased nutrient availability. They all synergistically increase the activity of the GLP1 producing neurons (termed PPG, or PPG neurons) in the NTS (81, 82).

The PPG NTS neurons have a wide projection field and synapse directly onto the VTA and NAc (83). Intestinally released GLP1 does not appear to cross the blood–brain barrier (BBB), and GLP1 receptor (GLP1R) signaling in the midbrain and basal forebrain requires NTS-mediated release of GLP1 (84). Activating GLP1R decreases highly palatable food intake and produces long-term weight loss; conversely, inhibiting them increases food consumption (83). Both changes occur due to changes in meal size, not frequency, indicating their role in terminating meal consumption. GLP1R activity in the VTA reduces LTP formation on dopaminergic neuron (85). GLP1 administration significantly increases dopamine transporter expression, increasing dopamine clearance from the synapse (86). GLP1R activity in the reward system may act to place the brakes on dopaminergic response to nutrient contents in the gut and prevent excessive learning of a food reward—or any reward, given the wide applicability of the reward system pathway. Consistent with this prediction, GLP1R knockout mice exhibit enhanced reward learning on the conditioned place preference test and increased cocaine-induced locomotion—receptor agonism had the inverse effect (87, 88). Thus, the PPG NTS neurons clearly mediate a dopamine-dampening function in the reward system and use this as a mechanism to reduce the reward salience and value of food or other objects.

### POMC Neurons in the NTS and the Arcuate Nucleus

The role of POMC neurons is more complex, and much effort has been expended to explore their role in satiety. POMC is a precursor polypeptide which is cleaved by prohormone convertase 1 or 2 into α-, β-, or γ-melanocyte-stimulating hormone (MSH), corticotropin, and β-endorphin (89). These neuropeptides act *via* the MCR family, the best studied of which in regard to satiety are MC3R and MC4R. Deletion of MC4R produces obesity and hyperphagia; furthermore, the most common syndromic cause of obesity, Prader–Willi, occurs in part due to reduced cleavage of POMC (90, 91). The NTS and Arc POMC neurons both drive satiety *via* their actions on their receptors, but at different time scales. Optogenetic stimulation of the POMC NTS immediately halts feeding, but long-term activation does not produce weight loss (92). Stimulation of POMC Arc neurons has no immediate effect on feeding, but over many hours reduces feeding and produces weight loss (37). Both populations of POMC neurons project to the NAc and VTA—however, evidence is lacking on what effect direct stimulation of these projections produces (36). Both GABA-expressing and glutamate-expressing neurons have been found within the POMC neurons of the arcuate nucleus; but, the effects of fast neurotransmission from these neurons are not well explored (93). Instead, this discussion will focus on the pharmacological and receptor-related data that exist on the role of MC3R and MC4R in these regions.

### MCR Signaling

The pharmacological effects of MCR activation are complex and their signaling mechanisms require some discussion before elaborating on their influence on the reward system. Multiple endogenous ligands for melanocortins exist. AgRP is an inverse agonist of MC3R and MC4R. α-, β-, and γ-MSHs and adrenocorticotropin hormone are produced from POMC by alternative cleavage and have varying affinities for each MCR type. Their downstream signaling activity is significantly altered dependent on their heterodimerization with other receptors such as dopamine receptors or ghrelin receptors. MC4R can couple with G_s_, G_q_, or G_i/o_ protein complexes dependent upon allosteric binding-related conformational changes and thus produce increases in intracellular calcium, increased cAMP signaling, or inhibit these same pathways (33). The two neuron-activating pathways, G_s_ and G_q_, mediate distinct physiologic effects; knockout of PVH G_q_, but not G_s_, prevented the efficacy of MC4R agonism to prevent food intake (94). Conversely, the ghrelin receptor, when coupled with either MC3R or MC4R, selects for G_s_ signaling in both proteins in the PVH, for example, emphasizing that in conditions of increased hunger, MSH activity may select for activation (95). One of the most commonly used non-selective synthetic MCR agonists, melanotan II (MTII), also selects for G_s_ signaling (33, 95). AgRP, discussed above, is an inverse agonist of MC4R and reduces cAMP levels independent of the presence of any agonist (33–35). Hypothetically, AgRP activity may select for the G_i_ signaling conformation of MC4R, as it both antagonizes the ligand-binding site and binds to the allosteric site to cause a conformational change; it also induces receptor internalization and alters MC4R-mediated inhibition of L-type calcium channel activity (31–33, 96). While it has been demonstrated conclusively that intracerebroventricular infusion of the MC3R/MC4R agonist α-MSH suppresses feeding and infusion of AgRP suppresses feeding, the multiple signaling pathways invoked by each ligand as compared to MTII are important considerations while evaluating the pharmacological data for melanocortin influence on the reward system (97).

MC4R is found on D1R MSNs in the NAc. Application of α-MSH to *ex vivo* brain slices produces a decreased post-synaptic ratio of AMPA/NMDA glutamatergic receptor signaling and fewer excitatory post-synaptic currents—a sign of long-term depression (LTD) (98). These MC4R-linked D1R MSNs synapse onto LH GABAergic neurons; optogenetic activation of these prevents feeding (99). This reduction in feeding also occurs by MCR activity in the VTA; injection of MTII decreases sucrose consumption (100). Thus, MC4R activity may increase activity of these neurons to inhibit feeding, but lose their synaptic strength to prevent excessive inhibition of feeding behavior and simultaneously reduce their rewarding value. This coincides with evidence that administration of MC4R shRNA (knockdown) prevents the natural decrease in reward seeking induced by a chronic stress paradigm (98). Furthermore, blockade of NAc MC4R prevents chronic stress-elicited anhedonia, a known low-dopamine phenomenon (98).

However, in other conditions MCRs may play a role in reward-mediated learning and sensitivity to reward. After 2 h, intraventricular injection of MTII reduced the threshold for brain self-stimulation (101). Furthermore, mice with knockdown or inhibition of MC4R display reduced cocaine-induced reinforcement and reduced locomotor sensitization (102, 103). Injection of alpha-MSH into the posterior VTA increases the activity of MC4R-expressing dopaminergic neurons and induces ethanol self-administration (104). These neurons coexpress MC3R, and agonism of these neurons with γ-MSH increases motivation to work for sucrose reward in a dopamine-dependent fashion (105). Indeed, it appears that the level of glucose increase in response to food intake induces excitatory synaptic plasticity in a subpopulation of POMC neurons, which may enhance some of these downstream dopaminergic responses (106).

Thus, the multiple combinations of POMC cleavage products, the heterodimerization and allosteric-binding configurations of MCRs, and the differences in activity dependent on brain region all indicate that further evaluation of intracellular signaling pathways is needed. However, a possible synthesis may be as follows. MC4R signaling reduces ongoing feeding *via* its action on D1R MSNs in the NAc, with a naturally decaying signal because of the development of LTD. Simultaneously, in other regions and neuronal subpopulations, MC3R and MC4R enhance synaptic plasticity to encode future responses to that food reward—hence, knockdown of these receptors reduces reward consumption. The balance of melanocortin-mediated LTP and LTD in striatal neurons is important to halt ongoing food consumption, but appropriately encourage future responsiveness to rewarding food cues. One demonstration of this phenomena arises when hyperphagic MC4R knockout mice have induced second deletion of synapse-associated protein 90/post-synaptic density protein 95-associated protein 3 (SAPAP3) in the striatum (90). This double deletion both cures the compulsive grooming behavior of these mice and normalizes their weight. Deletion of SAPAP3 induces excessive LTD formation, and one may postulate that this helps reduce the excessive excitatory synapses formed in the VTA by unchecked MC3R signaling that in turn promote feeding behavior (107).

## Food-Related Hormones Influence Dopaminergic Activity and Sensitivity to Reward

The above discussion has focused on the interaction between neurons encoding hunger and satiety and the reward system. However, these are all interoceptive and are responsible for the integration of internal and external sensory information to produce unified behaviors. As is to be expected, the sensory input into this system is both neuronal (gut distention, pain, olfaction, taste, etc.) and endocrine. While the vast majority of endocrine pathways are altered in some way in response to variations in energy stores and the presence or absence of food in the gut, the best studied of these which appear to act directly on the reward system are the hunger hormone ghrelin and the satiety hormone leptin.

### Ghrelin

Ghrelin is a peptide hormone first discovered as an endogenous ligand of the growth hormone secretagogue receptor (GHS1R), produced in the oxyntic glands of the stomach (108). Copies of ghrelin mRNA and ghrelin immunoreactivity are found in abundance in the antrum and fundus of the stomach and to a lesser degree in the duodenum, jejunum, ileum, and pancreas (109–111). Both peripheral and intracerebroventricular injections of ghrelin produce feeding in mice, and knockout of neuronal GHS1R prevents the development of diet-induced obesity; this places the CNS activity of ghrelin as a primary mediator of ghrelin’s orexigenic effect (112, 113).

The mechanics governing ghrelin release and the neuronal response to ghrelin are complex and deserve some consideration. Ghrelin secretion occurs in ultradian pulses, peaking immediately before onset of meals and declining soon after, with the greatest rise occurring overnight preceding breakfast (114). Several neurotransmitters, hormones, and metabolic signals also affect ghrelin release; acetylcholine, CCK, gastrointestinal peptide, and low glucose concentration enhance it, whereas insulin, gastrin, somatostatin, GLP-1, and increasing glucose concentration inhibit its release (115–117). Once ghrelin is produced, it can undergo post-translational modification with fatty acid linkage (octanoylation). This appears to affect ghrelin transport across the BBB; human octanoylated ghrelin and mouse des-octanoyl ghrelin are preferentially transported into the brain, whereas mouse octanoylated ghrelin (with two amino acid differences) is preferentially transported into the blood (118). The rate of BBB transport is enhanced by elevated serum triglycerides and fasting and blunted in aging, indicating physiological state modulation of this mechanism (119). There is also some evidence suggesting that a subpopulation of ghrelin-producing neurons may exist within the hypothalamus itself, which would not be affected by BBB transport and would have entirely unique, as yet undescribed, regulation (38). Finally, GHS1R-Gq-induced calcium flux (i.e., the signal strength) is attenuated by its heterodimerization with serotonin (specifically 5-HT2C), dopamine (D1R), and melanocortin (MC3R) receptors (120). Given that GHS1R possesses one of the highest levels of basal Gq activity of any GPCR, this dimerization may be important for reducing basal signaling except in the presence of the appropriate ligands for each receptor, potentiating the signal strength of the ghrelin-GHS1R active conformation or increasing GHS1R Gq-signaling *via* dedimerization after dopamine-D1R or serotonin-5-HT2C interactions (121).

After integrating the influences of pulsivity, BBB transport, and heterodimerization considered, ghrelin induces feeding behavior by depolarizing neurons expressing GHS1R. Chief among these, AgRP/NPY neurons express GHS1R-G_q_-coupled signaling; this produces feeding behavior and reduced thermogenesis (112, 122). Thus, in the absence of any food cues, ghrelin actually reduces sensitivity to reward, acting *via* AgRP/NPY neurons and inhibiting the VTA and NAc as described earlier. However, GHS1R is also found in the VTA and laterodorsal VTA, a source of midbrain acetylcholine; injecting ghrelin into either of these places increases locomotor activity, food consumption, and NAc dopamine levels (123). Indeed, ghrelin enhances the rewarding value of high fat diet in *ad libitum*-fed mice (124). If no food is consumed after VTA ghrelin injection, VTA GABAergic neurons increase activity and reduce the release of dopamine into the NAc (123). These effects occur both due to ghrelin action on the VTA cell body and by alteration of pre-synaptic activity, especially the LH as orexin-deficient mice are resistant to the effect of ghrelin (40, 124–126).

These effects may be cross-sensitive for non-food rewards such as alcohol and amphetamines. In recovering alcoholics, ghrelin injection increases craving for alcohol; coincident with this, ghrelin receptor blockade attenuates both alcohol and amphetamine-induced locomotion sensitization (127–130). However, once sensitization has occurred, blockade of ghrelin transport into the brain does not appear to alter alcohol-induced locomotor activity or expression of conditioned place preference in rats (131). Thus, ghrelin may act to enhance learning of non-food rewards, but not be necessary to express this preference (132). Notably, GHS1R is found in the hippocampus; here it may enhance dopamine-induced synaptic plasticity within the hippocampus, promoting retention of the food-related reward *via* GHS1R-D1R heterodimer interactions (133).

Ghrelin action in gaging reward sensitivity can be summarized in three ways. First, by acting on AgRP/NPY neurons, it selects for food-seeking and food-consuming behavior and ignoring of other rewards. Second, by acting on the VTA, it increases the amplitude of dopamine release once cue-mediated silencing of AgRP/NPY neurons disinhibits these regions. Third, by acting on the hippocampus, it promotes dopamine-induced synaptic plasticity and future salience of the reward cue. Given the complexity and numerous levels of signal modification in the ghrelin system, future research will undoubtedly expand upon this story.

### Leptin

Leptin is a signal of positive energy balance and appears to exert a dampening effect on the reward system. Leptin is a protein hormone released by white adipose tissue in pulses, highest around midnight in humans; the amplitude of each pulse directly correlates with the total amount of adipose tissue (114, 134). Glucose, insulin, glucocorticoids, TNF-alpha, and IL-6 all increase leptin release (134). Leptin acts on its receptors (leptin receptor, LepR), which are located in the CNS, particularly the hypothalamus. It can influence neuronal activity from the blood *via* action on the circumventricular organs and vagal afferents and is also translocated across the BBB *via* the action of tanycytes (135). The Arc, ventromedial and dorsomedial hypothalamus, LH, and NTS most densely express LepRs, and leptin action can be either inhibitory or excitatory on these neurons to inhibit food consumption and induce satiety (136). LepR also exists within the VTA on the dopaminergic projections to the extended central amygdala; exciting these neurons reduces food intake (137–139). This is consistent with the central amygdala’s role in mediating the stress response.

Other populations of LepR expressing dopaminergic neurons have their activity inhibited by leptin and produce a general reward-dampening effect. Direct administration of leptin into the VTA and into the Arc increases the threshold for brain self-stimulation reward and decreases food intake (140). Conversely, knockdown or inhibition of LepRs within the VTA increases dopamine release onto the NAc and enhances cocaine-conditioned place preference (13). LepR depletion within the NAc core mediates the same effect (13). Interestingly, it also prevented D2R agonism from reducing cocaine reinforcement, implying that there is some synergy between LepR inhibitory action and D2R’s reduction of synaptic plasticity mechanisms (13). This may occur *via* LepR activation of the signal transducer and activator of transcription 3 signaling pathway in the VTA, which reduces both feeding behavior and motivation to exercise (141). Finally, the hyperleptinemia of diet-induced obesity is also sufficient to produce the generalized reward-dampening effects described above. Mice with diet-induced obesity have reduced drive to self-administer cocaine, express less amphetamine conditioned place preference, and are less likely to express operant response to sucrose (142, 143). These mice are demonstrated to have reduced dopamine turnover in the NAc (142, 143). Interestingly, alcohol consumption does not appear to follow this paradigm. Leptin administration significantly increased the consumption of ethanol in mice exposed to an ethanol deprivation-then-reinstatement procedure (144). High leptin levels at onset of alcohol withdrawal in humans were predictive of cravings and alcohol relapse; anti-opioid receptor drugs used to prevent relapse reduced leptin levels (145). Future research is necessary to understand how alcohol bypasses the dopaminergic reward system pathway to induce opioid release in a leptin-potentiated manner. Nevertheless, leptin clearly induces satiety by directly reducing activity of both the hunger system and the reward system, with generalizable reduction of the salience and value of both food and non-food rewards.

## Limitations in the Study of the Interface Between the Hunger and Reward Systems

The above reviewed evidence is not comprehensive, as the relationship between the hunger system and reward system is of great interest and under active research with exciting new evidence unveiled daily. Given the ancient association of these two systems and the necessity of proper balance of food consumption to sustain life, it is understandable that nearly every neuronal and endocrine system appears to alter food consumption and manipulate the reward system in some way. Furthermore, the developmental activity of these systems may differ from their adult activity, and disruptions to these systems may result in significant compensation over the developmental period. The techniques utilized to study feeding and reward behavior are also limited by the necessity of overactivation, for an extended period, a single node within the feeding system. Given the interconnected nature of these neuronal circuits within the hunger system, continuous activation of any part likely engages the other portions and produces the unified behavioral response. However, it is likely that each neuronal pathway described above does not independently induce or inhibit food or reward consumption, but instead activates a certain portion of a “reward consumption sequence.” A clear example of this is how LH-VTA GABAergic activation induces gnawing and licking behaviors, but not sucrose seeking (62). The nature of this reward consumption sequence remains to be uncovered, and will likely require further molecular characterization of each of these components of the reward sequence, *in vivo* recording of their activity, and more advanced optogenetic and chemogenetic stimulation techniques to better approximate physiological activity.

## Conclusion

The hunger system manipulates the reward system to increase the salience and value of food and enhance future response to rewarding food cues. Ghrelin and AgRP neurons prime the reward system to activate only in the presence of food cues. LH fast neurotransmitters and orexin neurons produce food-seeking paradigm in response to a learned cue. Other LH GABAergic neurons and MCH neurons increase food consumption, reducing reward seeking behavior and producing reward consuming behavior by their action on the VTA and NAc. Early satiety signals like GLP1 and CCK put the brakes on food consumption by dampening reward system activity, reducing its value. POMC neuronal activity produces a balanced synaptic plasticity to respond effectively to future food stimulus, while gradually inducing satiety. Late satiety signals like leptin dampen reward system activity generally to reduce effort for acquiring food or other rewards while in a positive energy state. A summary of these effects is presented in Table 1.

**Table 1:** **Hunger system activity and influence on the reward system**.

	Fasting state	Food cue response	Feeding and fed state
Arcuate agouti-related protein (AgRP)/neuropeptide Y neurons	Inhibits reward system activity	Decreased activity	Decreased activity
Lateral hypothalamic area (LH) GABAergic neurons	Unknown	Inhibit subpopulation ventral tegmental area (VTA) DA and GABAergic neurons	Inhibit different subpopulation of VTA DA and GABA neurons
LH Orexin neurons	Increase reward system sensitivity	Increase VTA DA firing	Reduced activity as glucose increases
LH melanin-concentrating hormone neurons	Inhibited in low glucose	Integrate gustatory cues, enhance nucleus accumbens (NAc) sensitivity	Activate in response to gustatory nutrient content, enhance NAc-mediated reward consumption
Nucleus tractus solitarius (NTS) preproglucagon neurons	Decreased activity	Decreased activity	Integrates cholecystokinin, leptin, glucagon-like peptide 1, gut distention to inhibit reward system
NTS pro-opiomelanocortin (POMC) neurons	Decreased activity	Decreased activity	Increase in response to gut distention, reduce feeding
Arcuate POMC neurons	Decreased activity	Decreased activity	Activated by leptin, decrease NAc sensitivity but increase VTA encoding of food cue
Ghrelin	Dual action: increases AgRP neuronal activity and increases intrinsic reward system sensitivity	Increases reward system sensitivity	Reduces after food consumption
Leptin	Decreased activity	Decreased activity	Increases with insulin activity and decreases reward system activity

With this paradigm in mind, we can return to the superstimulus concept and observe how extreme rewards may bypass this balanced relationship of hunger systems enhancing food rewards to increase food consumption, and satiety systems reducing food rewards to reduce consumption. Highly palatable foods can induce food consumption in fed mice, even with adult ablation of AgRP neurons—a disruption which typically produces starvation (146). This relies on dopaminergic tone to produce the feeding response. A natural extension of this finding is this: if the reward’s learned value is high enough to stimulate a large dopamine spike upon sighting, overcoming the reward-dampening influence of satiety circuits and hormones, this triggers the feeding sequence. It can be speculated that normally, AgRP neurons *via* their aversive, inhibitory action sensitize the reward system so that when it is finally released, even an intrinsically low-value food cue produces a large spike in dopaminergic activity. This is in keeping with the old saying that “hunger is the best sauce.” Thus, it may not be that there are separate hedonic and homeostatic feeding mechanisms, but instead that highly palatable foods bypass the natural inhibition of the reward system by the hunger system.

## Author Contributions

RC prepared and wrote the manuscript. QT revised the manuscript and provided critical input for new ideas and content to be added to the manuscript.

## Conflict of Interest Statement

The authors declare that the research was conducted in the absence of any commercial or financial relationships that could be construed as a potential conflict of interest.

## Abbreviations

AgRP, agouti-related protein; Arc, arcuate nucleus of the hypothalamus; CCK, cholecystokinin; D1R, D2R, dopamine receptors; GHS1R, growth hormone secretagogue 1 receptor (ghrelin receptor); GLP1/GLP1R, glucagon-like peptide 1/receptor; LepR, leptin receptor; LH, lateral hypothalamic area; LTD, long-term depression; LTP, long-term potentiation; MC3R, MC4R, melanocortin receptors; MCH/MCH1R, melanin concentrating hormone/receptor; MSH, melanocyte-stimulating hormone (natural MCR agonist); MSN, medium spiny neuron; MTII, melanotan II (non-selective MCR agonist); NAc, nucleus accumbens; NAcc, nucleus accumbens core; NAcSh, nucleus accumbens shell; NTS, nucleus tractus solitarius; O1R, O2R, orexin receptors; POMC, pro-opiomelanocortin; PPG, preproglucagon; PVH, paraventricular hypothalamic nucleus; VTA, ventral tegmental area.
